# Diversity in German-speaking medical ethics and humanities

**DOI:** 10.1007/s11673-022-10215-6

**Published:** 2022-11-07

**Authors:** Amelia Fiske, Stuart McLennan

**Affiliations:** Institute of History and Ethics in Medicine, TUM School of Medicine, Technical University of Munich, Ismaninger Straße 22, 81675 Munich, Germany

**Keywords:** Medical ethics, Medical humanities, Germany, Switzerland, Austria, Diversity, Gender, Race, Academia

## Abstract

**Background:**

Bioethics can play an important role in addressing diversity both in and outside of academia, setting precedents for meaningful contributions to public discourse, research, teaching, training, and policy development. However, in order to do so, these conversations also need to reflect on the issue of diversity within the field of bioethics across the globe. This study aims to examine current gender representation and diversity at medical ethics and humanities institutes in Germany, the German-speaking areas of Switzerland, and Austria.

**Methods:**

A total of forty-nine medical ethics and humanities institutes from Germany (n=42), the German-speaking areas of Switzerland (n=5), and Austria (n=2) were included in the study. Institutes websites were reviewed in the first week of March 2021 and the details of each staff member listed on the website recorded.

**Results:**

Overall, a total of 964 staff members were identified at the forty-nine German-speaking medical ethics and humanities institutes. Just over half (530/964; 55%) of all staff were female. There were significant differences between gender in some staff positions: 64.6 per cent (31/48) of directors were male (χ^2^_(1)_=4.1, *P=*.04); 62.7 per cent (84/134) of student assistants were female (χ^2^_(1)_=8.6, *P=*.003); and 83.7 per cent (77/92) of administrative staff were female (χ^2^_(1)_=41.8, *P*<.001). There were no significant differences between staff gender for researchers and lecturers, or associated researchers. In addition, 65.5 per cent (19/29) of researchers and lecturers who had a professor title were male, but the difference between genders was not found to be significant. However, significantly more of the researchers and lecturers who had completed a habilitation were male (75.8% (25/33); χ^2^_(1)_=8.8, *P=*.003). When comparing the institute director’s gender presentation with staff gender presentation, it was found that male-led institutes had 53.4 per cent (286/536) female staff overall but had 52.7 per cent (136/258) male researchers and lecturers. However, the difference between genders were not found to be significant. On the other hand, female-led institutes had significantly more female staff overall (59.9% (223/372); χ^2^_(1)_=14.7, *P*<.001) and also significantly more female researchers and lecturers (58.9% (119/202; χ^2^_(1)_=6.4, *P=*.01).

**Conclusions:**

There has been a significant push to address gender diversity in German-speaking academia, and this study finds overall good gender parity in medical ethics and humanities institutes. However, there has not been a similar openness to discussing issues of systemic racism or how other forms of inequality affect academic diversity. Taking diversity seriously requires opening up conversations around intersectionality, including difficult conversations around race and cultural background that have long been taboo in German-speaking countries.

**Supplementary Information:**

The online version contains supplementary material available at 10.1007/s11673-022-10215-6.

## Introduction

Historically focused on clinically oriented concerns, bioethicists have shied away from strong positions on social justice despite mounting calls to do so. However, recent events, including the persistent effects of gender inequality and racism in health disparities, are bringing about a reckoning for the discipline, pointing to the collective failure to forcefully combat structural inequality (Association of Bioethics Program Directors 2020). Movements such as #BlackBioethics and #LatinXBioethics have underscored the need for bioethics to tackle critical health issues such as institutional racism, LGBTQAI+ ethics, and social justice (Mayes, Paradies, and Elias 2021; Wilson 2021; Truong and Sharif 2021; Klugman 2017). The tendency of mainstream bioethics to “problematize and displace” rather than focus on key issues such as diversity, serves to reproduce the status quo in bioethics (Myser 2003). In particular, the issues disproportionately affecting women, queer and nonbinary people, and people of colour have not been centred in mainstream bioethics. As Keisha Ray recently argued, the field’s bias in this respect reflects an institutional blind eye to the needs, scholarship, and labour of marginalized groups (Ray 2020). This tendency extends beyond issues of scholarship alone, calling for a critical look at the composition of bioethics institutes across the globe.

In relation to diversity, conversations in German-speaking countries of Germany, Switzerland, and Austria have largely hinged on issues of gender and, to a lesser extent, disability. As such, we begin our exploration into questions of representational diversity in German-speaking medical ethics and humanities institutes by focusing on gender inequality. Following the World Health Organization, we understand gender inequality to refer to both matters of equality (the absence of discrimination on the basis of sex in opportunities, the allocation of resources and benefits, or access to services) and equity (fairness and justice in the distribution of benefits and responsibilities between women and men) in academia (WHO 2022). Gender inequality in academia is a multifaceted and complex issue shaped by structural inequalities result in uneven patterns of inclusion (Fitzgerald 2007).

A growing body of international research has sought to probe the highly contested and complex issue of gender inequality, which includes matters of rights, attitudes, practices, resource distribution, and capabilities (Krzaklewska 2014; Holter, Svare, and Egeland 2009; Neyer, Lappegård, and Vignoli 2013). Feminist researchers have long maintained that academia is both hierarchical and discriminatory (Powell, Ah-King, and Hussénius 2018; Benschop and Brouns 2003; Bacchi and Eveline 2010; van den Brink and Benschop 2012), noting the many ways in which the academy is organized by, and actively maintains, hierarches of class, race, and nation, and reproduces racialized and gendered inequalities (Ferree and Zippel 2015). Institutional efforts seeking to address gender equality in the academy have faced significant challenges, often failing to effect deep-seated change and experiencing resistance from other staff (Powell, Ah-King, and Hussénius 2018; Ferree and Zippel 2015). As Kreissl et al. illustrate, this can also include institutional structures that are difficult to shift (2015). Research on efforts to improve gender equality has illustrated the problems of tokenism and the risks of “treating diversity as a box to check” (Chang and Milkman 2020) without addressing issues such as pay gaps (Blau and Kahn 2017; Flabbi et al. 2019). Even when adequate levels of gender parity are achieved, women often face additional challenges, such as exclusionary practices, unfair departmental workload distribution, unconscious bias, and expectations that they adopt stereotypically “male” traits of success in order to be professionally successful (Peterson and Jordansson 2017; Peterson 2015).

Equal opportunity programs at German universities began in the 1990s, and gender representation in higher education has been tracked since 1997. In June 2020, the German federal government unveiled a national strategy for gender equality, including the establishment of a federal foundation for gender equality to address goals such as equal pay and pensions and improving career opportunities for women (Colbourne 2020). While women continue to be under-represented in the highest levels of the academy and in leadership positions, their participation is steadily improving. At the start of university level studies, men and women are equally represented; however, a significant decline in women’s participation occurs in the transition to the doctoral and postdoctoral levels (GESIS 2020a). At present, a little over one-third (37%) of new appointments to professorships are women; however, it is estimated that it will take another thirty years for women to reach gender parity at the highest level of tenured professor in Germany (“W3”) (GESIS 2020b). The number of women receiving their habilitation, a qualification traditionally required for becoming a full professor following the completion of the doctorate, has increased more than six-fold since 1980. In 2019, 31.9 per cent of habilitations were held by women (GESIS 2020b). However, in recent years, new paths to professorship for younger scholars have been created such that the habilitation is not always required. Such routes may help to increase gender representation at the level of professors in Germany.

In Switzerland, equal opportunity measures were enacted in 1981, guaranteeing men and women equal opportunities and remuneration. By 2000, momentum was building with the initiation of the “Federal Programme for Equal Opportunities for Women and Men at Universities,” accompanied with substantial government investment aimed at increasing appointments of female professors, promoting young scientists, and creating gender monitoring programmes (Herrmann et al. 2019). In the past decades, progressively more women have entered into university programmes (Leybold-Johnson 2017). Women now comprise 41 per cent of those completing doctorates, 40 per cent of non-tenured, scientific collaborators, and 18 per cent of professors; however, it is worth noting that the number of female professors has improved dramatically from a mere 2 per cent in 1980 (Dubois-Shaik and Fusulier 2015). Conversations around diversity have typically hinged on gender, while matters such as race are often not considered because they are not seen as central issues in Switzerland (Nentwich 2006).

There has also been a steady increase in women’s participation in the higher education landscape in Austria (Pellert and Gindl 2007; Wroblewski and Leitner 2011). Nonetheless, women’s participation remains higher among administrative positions than academic ones. Despite significant gains since the 2000s, women comprise approximately forty per cent of PhD level graduates, thirty per cent of those working as scientists and researchers, and twenty per cent of professorships (Wroblewski and Striedlinger 2018). The government agenda in Austria for 2013–2018 included specific goals relating to the promotion of women’s advancement in higher education and leadership (European Institute for Gender Equality 2021), and more than a third of all research institutions have enacted gender equity plans (Wroblewski and Striedlinger 2018).

The need for better representation of women in higher levels of academia is a widely recognized international concern (Harford 2018). The European Commission’s most recent report on gender inequality in 2021 also found that women represented approximately one-third (32.8%) of researchers at the European level, with little change since 2015, suggesting that despite political commitments to gender equality (e.g., directives addressing precarious work, work-life balance, digital skills, and decision-making and leadership) that there has been little change (European Commission 2021, 96). Women’s participation in academia declines in relation to leadership and hierarchy across the EU. While women were well represented in BA and MA programmes (54% students and 59% graduates) and held nearly half of grade C staff positions (47%), their representation declined to around a quarter (26.2%) of the highest level of staff (grade A, equivalent to full professorship positions). On the whole, these trends were generally better in the humanities and social sciences and worse in STEM fields (European Commission 2021).

Efforts to address gender inequality and diversity in the academy currently range from a focus on equal rights to individual attitudes, the distribution of resources, shared decision-making, power relations, and hidden discrimination (Krzaklewska 2014). Research on gender inequality thus requires a multidimensional approach that is oriented towards the intersectionality of different forms of structural inequality, including race, class, migration-background, ability, and more (Holter, Svare, and Egeland 2009; Grzanka et al. 2016). As the above review suggests, there has therefore been a significant push to address gender diversity in German-speaking academia; however, there has not been a similar openness to an intersectional approach to diversity, such as examining how gender inequality intersects with issues of systemic racism or how other forms of inequality affect academic diversity. To our knowledge, there are no available data on staff diversity at German-speaking medical ethics and humanities institutes in terms of gender, people of colour, those coming from diverse immigration backgrounds, those of queer or nonbinary gender identifications, or other marginalized groups. As such, it is not possible to formally assess if improvements have been made in cultivating diverse research environments in the past decades.

Bioethics can play an important role in addressing diversity both in and outside of academia, setting precedents for meaningful contributions to public discourse, research, teaching, training, and policy development (Danis, Wilson, and White 2016). However, in order to do so, these conversations also need to reflect on the issue of diversity within the field of bioethics across the globe. In order to support and open conversations surrounding what diversity in bioethics might mean in the German-speaking academic context, this study aims to take a first step towards examining current representational gender diversity at medical ethics and humanities institutes in Germany, the German-speaking areas of Switzerland, and Austria. We recognize that one-dimensional approaches to questions of complex inequalities are insufficient. Thus, we proceed with the caveat that gender inequality is never experienced in isolation and is always co-constituted by systemic inequalities (Grzanka et al. 2016). Given the dearth of information available on matters of diversity within the field of bioethics in German-speaking countries, in this initial study we aim to open conversations about who is “sitting at the table” in the field. We use this as a starting point to reflect on some of the systemic problems relating to intersectionality and diversity in relation to leadership and research agenda in the field and the need to broaden research into questions relating to matters of race, migration-background, and membership with other marginalized groups, as well as diversity of research agendas within institutes, lived experiences of marginalization in academia, and more.

In the spirit of reflexivity, and the necessity of considering one’s own positionality in relation to highly politicized issues of diversity, we would like to situate ourselves in relation to this scholarship before beginning. A.F. is a white woman originally from the United States. She holds a PhD in cultural anthropology, with a specialization in medical anthropology, from the University of North Carolina at Chapel Hill. She has lived and worked in Germany for the past six years as a researcher in medical ethics and humanities. She is currently a senior researcher at the Technical University of Munich; she has previously worked at the University of Kiel and taught medical ethics and humanities in Austria. S.M. is a white man originally from New Zealand. He has lived in Germany for thirteen years, and recently became a naturalized German citizen. He holds a PhD and habilitation in biomedical ethics from the University of Basel in Switzerland. He is currently a director of research at the Technical University of Munich and teaches medical ethics and humanities in Austria; he has previously worked at the Ruhr University of Bochum and the Hannover Medical School. This research is inspired by conversations we have had with others in the field on matters of race, gender, and representation within academia, and our own experiences of relative privileges of whiteness and education as foreigners working within this system.

## Methods

### Sample

Institutes conducting research and teaching regarding medical ethics and humanities in Germany, the German-speaking areas of Switzerland, and Austria were included. Institutes were primarily identified via the German Academy of Ethics in Medicine’s (Akademie für Ethik in der Medizin e.V. [AEM]) list of scientific institutes for ethics in medicine in Germany, Switzerland, and Austria (Akademie für Ethik in der Medizin 2021). However, the Saarland University´s Institute of Human Genetics was excluded; although the AEM’s list notes that the institute´s work covers ethics, no ethics research or teaching could be identified on its website, and we felt that it did not make sense to include a human genetics institute in a study focused on medical ethics and humanities. In addition, a number of other relevant institutes or medical ethics and humanities working groups known by the authors or identified by searches on Google, but not on the AEM’s list of institutes, were included. Overall, a total of forty-nine institutes from Germany (n=42), the German-speaking areas of Switzerland (n=5), and Austria (n=2) were included in the study.

### Data Collection and Analysis

In the first week of March 2021, we reviewed the websites of all forty-nine institutes. For each institution, the details of each staff member listed on the website were recorded in a SPSS file (version 27, IBM Corp. Armonk, NY, USA). It was recorded whether the staff member’s position was the director of the institute or leader of the working group (when situated in a non-medical ethics or humanities institute or department), a researcher or lecturer at the institute (including all “wissenschaftliche*r Mitarbeiter*in”—professors, research fellows and assistants, and lecturers), associated researchers (including those listed as “associated researchers,” freelancers, emeritus professors, and PhD and master’s students with no listed research assistant position), student assistants (*wissenschaftliche Hilfskraft*), or administrative positions (including secretaries, IT support staff, and library staff). In addition, we recorded how many researchers and lecturers had a professor title or had completed a habilitation (a qualification usually required for independent teaching and to obtain a professorship). We also recorded each staff member´s presumed gender presentation on the basis of their photograph and/or name (male, female, or non-binary). Descriptive statistics included absolute and relative frequencies per category. To analyse differences between genders, chi-square tests were performed with a significance level alpha set to .05, using Statistical Package for the Social Sciences (SPSS version 24 for Windows, IBM Corporation).

## Results

A total of 964 staff members were identified at the forty-nine German-speaking medical ethics and humanities institutes (table 1). Overall, there were significantly more female staff (530/964; 55%; χ^2^_(1)_=9.6, *P=*.002). There were also significant differences between gender in some staff positions: 64.6 per cent (31/48) of directors were male (χ^2^_(1)_=4.1, *P=*.04), 62.7 per cent (84/134) of student assistants were female (χ^2^_(1)_=8.6, *P=*.003), and 83.7 per cent (77/92) of administrative staff were female (χ^2^_(1)_=41.8, *P*<.001). There were no significant differences between staff gender for researchers and lecturers (52.4% (241/460) female; χ^2^_(1)_=1.1, *P=*.31), or associated researchers (51.7% (119/230) males; χ^2^_(1)_=.28, *P=*.60). In addition, 65.5 per cent (19/29) of researchers and lecturers who had a professor title were male, but the difference between genders was not found to be significant (χ^2^_(1)_=2.8, *P=*.09). However, significantly more of the researchers and lecturers who had completed a habilitation were male (75.8% (25/33); χ^2^_(1)_=8.8, *P=*.003). When comparing the institute director’s gender presentation with staff gender presentation (table 2), it was found that male-led institutes had 53.4 per cent (286/536) female staff overall but had 52.7 per cent (136/258) male researchers and lecturers. However, the difference between genders in these positions was not found to be significant. On the other hand, female-led institutes had significantly more female staff overall (59.9% (223/372); χ^2^_(1)_=14.7, *P*<.001) and significantly more female researchers and lecturers (58.9% (119/202; χ^2^_(1)_=6.4, *P=*.01).

**Table 1:** Staff position vs staff gender

Country	Gender	Staff position n (%)					Total N (%)
		Director	Researchers and lecturers	Associated researchers	Student assistants	Admin	
Germany	Male	29 (69)	181 (47.6)	89 (54.6)	49 (36.8)	13 (15.7)	361 (45.1)
	Female	13 (31)	199 (52.4)	74 (45.4)	84 (63.2)	70 (84.3)	440 (54.9)
	Total	42 (100)	380 (100)	163 (100)	133 (100)	83 (100)	801 (100)
	Chi-square	χ^2^_(1)_=6.1, *P*=.01	χ^2^_(1)_=.85, *P=*.36	χ^2^_(1)_=1.4, *P=*.24	χ^2^_(1)_=9.2, *P*=.002	χ^2^_(1)_=39.1, *P*<.001	χ^2^_(1)_=7.8, *P*=.005
Switzerland	Male	2 (40)	38 (47.5)	26 (44.1)	1 (100)	2 (22.2)	69 (44.8)
	Female	3 (60)	42 (52.5)	33 (55.9)	0	7 (77.8)	85 (55.2)
	Total	5 (100)	80 (100)	59 (100)	1 (100)	9 (100)	154 (100)
	Chi-square	χ^2^_(1)_=.20, *P*=.65	χ^2^_(1)_=.20, *P*=.65	χ^2^_(1)_=.83, *P*=.36	N/A	χ^2^_(1)_=2.8, *P*=.09	χ^2^_(1)_=1.6, *P*=.19
Austria	Male	0	0	4 (50)	0	0	4 (44.4)
	Female	1 (100)	0	4 (50)	0	0	5 (55.6)
	Total	1 (100)	0	8 (100)	0	0	9 (100)
	Chi-square	N/A	N/A	χ^2^_(1)_=.000, *P=*1.0	N/A	N/A	χ^2^_(1)_=.11, *P*=.74
Overall	Male	31 (64.6)	219 (47.6)	119 (51.7)	50 (37.3)	15 (16.3)	434 (45)
	Female	17 (35.4)	241 (52.4)	111 (48.3)	84 (62.7)	77 (83.7)	530 (55)
	Total	48 (100)	460 (100)	230 (100)	134 (100)	92 (100)	964 (100)h
	Chi-square	χ^2^_(1)_=4.1, *P*=.04	χ^2^_(1)_=1.1, *P*=.31	χ^2^_(1)_=.28, *P*=.60	χ^2^_(1)_=8.6, *P*=.003	χ^2^_(1)_=41.8, *P*=.001	χ^2^_(1)_=9.6, *P*=.002

**Table 2:** German and Swiss director gender vs staff gender

Country	Director gender	Researchers and lecturers			Associated researchers			Student assistants			Admin			Staff overall		
		Males	Females	Chi-square	Males	Females	Chi-square	Males	Females	Chi-square	Males	Females	Chi-square	Male	Female	Chi-square
Germany	Male 29/42 (69)	130/248 (52.4)	118/248 (47.6)	χ^2^_(1)_=.58, *P*=.45	63/119 (52.9)	56/119 (47.1)	χ^2^_(1)_=.41, *P=*.52	30/88 (34.1)	58/88 (65.9)	χ^2^_(1)_=8.9, *P*=.003	11/58 (19)	47/58 (81)	χ^2^_(1)_=22.3,*P*<.001	234/513 (45.6)	279/513 (54.4)	χ^2^_(1)_=3.9, *P*=.05
	Female 13/42 (31)	51/132 (38.6)	81/132 (61.4)	χ^2^_(1)_=6.8, *P*=.009	26/44 (59.1)	18/44 (40.9)	χ^2^_(1)_=1.5, *P*=.23	19/45 (42.2)	26/45 (57.8)	χ^2^_(1)_=1.1, *P*=.30	2/25 (8)	23/25 (92)	χ^2^_(1)_=17.6,*P*<.001	98/246 (39.8)	148/246 (60.2)	χ^2^_(1)_=10.2,*P*=.001
Switzerland	Male 2/5 (40)	6/10 (60)	4/10 (40)	χ^2^_(1)_=.40, *P*=.53	8/10 (80)	2/10 (20)	χ^2^_(1)_=3.6, *P*=.06	1/1 (100)	N/A	N/A	1/2 (50)	1/2 (50)	χ^2^_(1)_=.000, *P*=1.0	16/23 (69.6)	7/23 (30.4)	χ^2^_(1)_=3.5, *P*=.06
	Female 3/5 (60)	32/70 (45.7)	38/70 (54.3)	χ^2^_(1)_=.51, *P=*.47	18/49 (36.7)	31/49 (63.3)	χ^2^_(1)_=3.4, *P=*.06	N/A	N/A	N/A	1/7 (14.3)	6/7 (85.7)	χ^2^_(1)_=3.6, *P=*.06	51/126 (40.5)	75/126 (59.5)	χ^2^_(1)_=4.6, *P*=.03
Overall	Male 31/47 (66)	136/258 (52.7)	122/258 (47.3)	χ^2^_(1)_=.76, *P*=.38	71/129 (55)	58/129 (45)	χ^2^_(1)_=1.3, *P*=.25	31/89 (34.8)	58/89 (65.2)	χ^2^_(1)_=8.2, *P*=.004	12/60 (20)	48/60 (80)	χ^2^_(1)_=21.6,*P*<.001	250/536 (46.6)	286/536 (53.4)	χ^2^_(1)_=2.4, *P*=.12
	Female 16/47 (34)	83/202 (41.1)	119/202 (58.9)	χ^2^_(1)_=6.4, *P*=.01	44/93 (47.3)	49/93 (52.7)	χ^2^_(1)_=.27, *P=*.60	19/45 (42.2)	26/45 (57.8)	χ^2^_(1)_=1.1, *P=*.30	3/32 (9.4)	29/32 (90.6)	χ^2^_(1)_=21.1,*P*<.001	149/372 (40.1)	223/372 (59.9)	χ^2^_**(1)**_=14.7, *P*<.001

Germany had 85.7 per cent (42/49) of all medical ethics and humanities institutes and had 83.1 per cent (801/964) of all staff (table 3). Overall, German institutes had significantly more female staff (54.9% (440/801); χ^2^_(1)_=7.8, *P=*.005). There were also some significant differences between gender in various staff positions: 69 per cent (29/42) of directors were male (χ^2^_(1)_=6.1, *P=*.01), 63.2 per cent (84/133) of student assistants were female (χ^2^_(1)_=9.2, *P=*.002), and 84.3 per cent (70/83) of administrative staff were female (χ^2^_(1)_=39.1, *P*<.001). There were no significant differences between genders in the positions of researchers and lecturers (52.4% (199/380) female; χ^2^_(1)_=.85, *P=*.36) or associated researchers (55.6% (89/163) male; χ^2^_(1)_=1.4, *P=*.24). German medical ethics and humanities institutes also had 93.1 per cent (27/29) of researchers and lecturers who had a professor title, and 81.8 per cent (27/33) of researchers and lecturers who had completed a habilitation. Although there was no significant difference between genders with regards to professor titles (66.7% (18/27) male; χ^2^_(1)_=3.0, *P=*.08), there were significantly more males with a habilitation (77.8% (21/27); χ^2^_(1)_=8.3, *P=*.004). Male-led German institutes had significantly more female staff overall (54.4% (279/513); χ^2^_(1)_=3.9, *P=*.05), and although 52.4 per cent (130/248) of researchers and lecturers were male, this was not significant (χ^2^_(1)_=.58, *P=*.45). Female-led German institutes had significantly more female staff overall (60.2% (148/246); χ^2^_(1)_=10.2, *P=*.001) and significantly more female researchers and lecturers (61.4% (81/132); χ^2^_(1)_=6.8, *P=*.009).

The five medical ethics and humanities institutes in Switzerland had a total of 154 staff members (table 4); 55.2 per cent (85/154) of staff were female, but there was no significant difference between genders (χ^2^_(1)_=1.6, *P=*.19). There were also no significant differences between genders in relation to any staff positions: 60 per cent (3/5) of directors were female (χ^2^_(1)_=.20, *P=*.65), 52.5 per cent (42/80) of researchers and lecturers were female (χ^2^_(1)_=.20, *P=*.65), 55.9 per cent (33/59) of associated researchers were female (χ^2^_(1)_=.83, *P=*.36), the one student assistant identified was male, and 77.8 per cent (7/9) of administrative staff were female (χ^2^_(1)_=2.8, *P=*.09). Only two researchers and lecturers had a professor title (one male and one female), and six researchers and lecturers had completed a habilitation (4/6; 66.7% male χ^2^_(1)_=.67, *P=*.41). Male-led Swiss institutes had 69.6 per cent (16/23) male staff overall and had 60 per cent (6/10) male researchers and lecturers. However, there were no significant differences between genders. Female-led Swiss institutes had significantly more female staff overall (59.5% (75/126); χ^2^_(1)_=4.6, *P=*.03), and although 54.3 per cent (38/70) of researchers and lecturers were female, this was no significant difference between genders (χ^2^_(1)_=.51, *P=*.47).

The two medical ethics and humanities institutes in Austria listed nine staff members, 55.6 per cent (5/9) of whom were female (table 5). One only listed one female director and no other staff members, and the other was a network of institutions for medical ethics education with eight associated researchers (50% male, 50% female) and no clear director.

## Discussion

Our review of gender diversity at German-speaking medical ethics and humanities institutes has found overall good gender parity between male and female staff members, but that gender parity reduced relative to position. There were more men in directorship positions, more men with professor titles, and three-quarters of those who had completed a habilitation were men. Concurrently, there were more women in student assistantships, and administrative roles were also held predominantly by women. Such a pattern suggests that while gender representation at German-speaking medical ethics and humanities institutes is overall quite good, that there may be important structural factors affecting female-identifying researchers from advancing through academic hierarchies and achieving necessary titles, such as the habilitation, in order to become a professor or director. Female-led institutions tended to have higher rates of female staff overall and more female researchers and lecturers, suggesting that female directors can have a positive effect on gender parity in relation to institutional hierarchy.

While conducting the review of gender presentation across the institutes, we observed that although gender parity is reasonably good, bioethics remains an overwhelmingly white-presenting discipline in German-speaking countries. As many social theorists have demonstrated, race is an ever-shifting social construct and a political category, with material consequences, that is a product of colonial encounters (Golash-Boza 2016; Crenshaw et al. 1995). It is not possible to know what race “looks like.” As such, it is not possible for us to present data on racial diversity in the field without engaging in a much more in-depth investigation of the topic. This research is absolutely necessary but remains beyond the scope of the methods engaged in this paper. The same is true for other important areas of diversity, such as immigration background, Indigenous communities, queer or nonbinary gender identifications, age, or membership with other marginalized groups.

As our review shows, diversity can never be measured by a single metric. In this case we found important differences between overall staff representation and gender representation at levels of researcher, professor, and those holding a habilitation. Likewise, questions of diversity are far greater than issues of gender alone, extending to a need for more researchers of different racial and ethnic identifications, religions, background, abilities, and experiences in the field, with an attention to how a variety of experiences and backgrounds intersects with questions of hierarchy, position, and opportunity. Taking diversity within bioethics seriously requires opening up conversations around intersectionality, including conversations around race and cultural background. Such conversations can be particularly difficult in Germany, where the issue of race and ethnicity have long been taboo. Indeed, in our collective nineteen plus years living and working in Germany, it has not been uncommon to find ourselves seated at department or disciplinary meetings where everyone in the room is white-presenting. This whiteness is rarely acknowledged, for reasons which are, in part, sociohistorical. Unlike other places, the modifier “white” is rarely used, reflecting the way in which whiteness continues to operate as an unmarked, privileged category. For historical reasons stemming from the Second World War, Germany does not collect data on race or ethnicity (Oltermann and Henley 2020), meaning that it is impossible to know how many people of colour are employed in academia. The consequences of this have been noted by scholars researching race in education in Germany: “The German word for ‘race’ (*Rasse*) was removed from both popular and official usage following the Holocaust, but the colorblind lacuna this created has done little more than provide space for systemic racism while making it very difficult to name, track, or condemn” (Moffitt 2020). This “race taboo” poses particular challenges, in part because there is no common language for discussing race as a social construct.

Working on diversity in academia requires going beyond merely changing the demographics of the profession through proactive training and hiring practices to addressing the range of issues on bioethics research agendas. As Myser notes, one effect of the “normativity of whiteness” within bioethics is the potential to marginalize scholars (disproportionately bioethicists of colour) who address these issues (Myser 2003). It is the responsibility of the profession as a whole to consider the legacies of marginalization of scholarship by people of colour and minority groups: the perspectives of diverse scholars should not merely be perceived in contrast to dominant views without questioning the normative weight that dominant views have held (Myser 2003). In order for bioethics to take up diversity, the issues that matter to marginalized groups need to be given more prominence in research agendas. By extension, our review suggests that just as it matters who leads the institutes, representation at all levels of hierarchy is likely also critical for shifting research agendas and institutional priorities.

What would a change in the bioethics research agenda look like in German-speaking institutes? At present, issues on health inequalities or migrant health disparities are not being addressed, or at least not in a widespread manner. The lack of representation in the research agendas of medical ethics and humanities institutes is important given the changing composition of countries like Germany. For example, Germany is home to 1.77 million refugees from places such as Syria, Iraq, Turkey, Eritrea, and Afghanistan, many of whom arrived in the last five years (Rayes 2021). Significant local and federal efforts have assisted with integration, education, housing, and more (Gluns 2018). Just as the fabric of society is changing, bioethics needs to be prepared to take up the issues that matter to diverse and marginalized groups in the German-speaking countries—and to have people with shared experiences sitting at the table when questions on refugee health, racial discrimination, violence, or health disparities are discussed in policy settings, academic conferences, and in university and medical school classrooms. As the faces and experiences of German-speaking societies are shifting, bioethics needs to rise to the occasion by making sure that the diversity of the German-speaking population is represented both in bioethical institutions and research agendas.

Our review suggests that tackling issues of inequality, privilege, and representatively in the academy cannot be a one-size-fits-all approach. In Germany, Switzerland, and Austria, this will require having hard conversations about what diversity means in relation to specific histories around genocide, racism, ethnicity, colonialism, queerness, and more in a changing society. It includes working towards a common language for what diversity means and why it matters. It requires identifying barriers to inclusion and exploring how different forms of inequality are perpetuated in and outside of the academy. These conversations need not be the same as those occurring in other places (Ray 2020), but they certainly have much to learn from one another.

### Limitations

This study has some limitations that should be considered when interpreting the results. First, it is possible that some institutes conducting research and teaching regarding medical ethics and humanities in German-speaking countries were not included in the study. We are confident, however, that the list of included institutes is reasonably comprehensive. Second, this study is based solely on the information presented on institutes’ websites in March 2021. It is possible that some websites are not up to date, and therefore do not accurately reflect the staff members (or their qualification) currently working at the institute. However, in our experience, institute websites are generally kept up to date, so we do not think that this would have affected our results in a significant way. Third, we collected the details of each staff member listed on the website for each institute; however, some individuals were listed on more than one institute’s website; this occurred within and across countries. Although this was not a widespread issue, the total number of identified staff members working at German-speaking medical ethics and humanities institutes includes some individuals more than once. Fourth, we fully recognize that issues of diversity are not reducible to one issue, nor understood by quantitative measures alone. This survey is a first step to understanding who is represented in these institutes, from which more in-depth research into diversity can be built upon. Further research—specifically that which is in-depth and qualitative, and probes beyond the male-female gender binary—into matters of gender inequality and diversity is needed in order to better understand these dynamics in the field of bioethics. Finally, we were unable to ask staff how they self-identify; the assessment was primarily made on the basis of assumed gender presentation in website photos. It is possible that some individuals’ professional photos do not reflect their gender identity or are outdated or that we have made mistakes in categorizing them. Further research is needed to establish how staff self-identify and to expand research into the representation of queer and non-binary gender identifications in academia.

## Conclusion

The intersecting effects of inequality and privilege and the relationship to health and health outcomes form critical ethical concerns that are central bioethical issues. A culture of silence only further upholds the status quo. We believe that a strong place to begin would be to start tracking diversity—not only along singular indicators such as gender parity across different levels of the profession but also intersecting indicators of race, class, immigration history, and more in bioethics and in academia more broadly. There is a need to proactively hire researchers, including women, queer, nonbinary individuals, people of colour, and those of diverse backgrounds who are committed to a research agenda that addresses bioethical questions around structural racism, inequality, and vulnerability, and to make sure that this research is not marginalized within institutional bioethics agendas. The creation of context-specific resources such as the #BlackBioethics Toolkit to open conversations around what diversity should look like in a changing society, and how it can be fostered, can be another strong step forward.

## Supplementary Information

Below is the link to the electronic supplementary material.

Supplementary file1 (DOCX 39 KB)

Supplementary file2 (DOCX 17 KB)

Supplementary file3 (DOCX 15 KB)

## References

[CR1] Akademie für Ethik in der Medizin. Wissenschaftliche Institute für Ethik in der Medizin. https://www.aem-online.de/index.php?id=28. Accessed September 21, 2021.

[CR2] Association of Bioethics Program Directors. 2020. ABPD statement on violence, COVID, and structural racism in American society. https://www.bioethicsdirectors.net/abpd-statement-on-violence-covid-and-structural-racism-in-american-society/. Accessed September 21, 2021.

[CR3] Bacchi C, Eveline J (2010). Mainstreaming politics: Gendering practices and feminist theory.

[CR4] Benschop Y, Brouns M (2003). Crumbling ivory towers: Academic organizing and its gender effects. Gender, Work & Organization.

[CR5] Blau F, Kahn L (2017). The gender wage gap: Extent, trends, and explanations. Journal of Economic Literature.

[CR6] Chang E, Milkman K (2020). Improving decisions that affect gender equality in the workplace. Organizational Dynamics.

[CR7] Colbourne, F. 2020. “Milestone”: Germany unveils plan to boost gender equality. *The Local*, July 8. https://www.thelocal.de/20200708/germany-unveils-plan-to-boost-gender-equality. Accessed November 12, 2020.

[CR8] Crenshaw K, Gotanda N, Peller G, Inglis C (1995). Critical race theory: The key writings that formed the movement.

[CR9] Danis M, Wilson Y, White A (2016). Bioethicists can and should contribute to addressing racism. American Journal of Bioethics.

[CR10] Dubois-Shaik, F., and B. Fusulier, eds. 2015. *Academic careers and gender inequality: Leaky pipeline and interrelated phenomena in seven European countries.* Gendering the academy and research: Combating career instability and asymmetries (GARCIA). https://eige.europa.eu/sites/default/files/garcia_working_paper_5_academic_careers_gender_inequality.pdf. Accessed September 21, 2021.

[CR11] European Commission, Directorate-General for Research and Innovation. 2021. *She figures. Gender in research and innovation: Statistics and indicators.* Publications Office. doi:10.2777/06090.

[CR12] European Institute for Gender Equality. 2021. *Gender equality in academia and research: Austria*. https://eige.europa.eu/gender-mainstreaming/toolkits/gear/legislative-policy-backgrounds/Austria. Accessed September 21, 2021.

[CR13] Ferree M, Zippel K (2015). Gender equality in the age of academic capitalism: Cassandra and Pollyanna interpret university restructuring. Social Politics.

[CR14] Fitzgerald T (2007). An absent presence: Women professors at the University of New Zealand 1911–1961. Journal of Educational Administration and History.

[CR15] Flabbi L, Macis M, Schivardi F, Moro A (2019). Do female executives make a difference? The impact of female leadership on gender gaps and firm performance. The Economic Journal..

[CR16] GESIS Leibniz Institut für Sozialwissenschaften. 2020a. Frauen- und Männeranteile im akademischen Qualifikationsverlauf, 2019 https://www.gesis.org/cews/unser-angebot/informationsangebote/statistiken/thematische-suche/detailanzeige/article/frauen-und-maenneranteile-im-akademischen-qualifikationsverlauf. Accessed September 21, 2021.

[CR17] GESIS Leibniz Institut für Sozialwissenschaften. 2020b. Frauenanteile an Habilitationen, Berufungen, Professuren und C4/W3-Professuren, 1980–2019 https://www.gesis.org/cews/unser-angebot/informationsangebote/statistiken/thematische-suche/detailanzeige/article/frauenanteile-an-habilitationen-berufungen-professuren-und-c4-w3-professuren. Accessed September 21, 2021.

[CR18] Golash-Boza T (2016). A critical and comprehensive sociological theory of race and racism. Sociology of Race and Ethnicity.

[CR19] Gluns, D. 2018. *Social assistance for refugees in Germany*. LoGoSO Research Papers Number 4, Free University Berlin.

[CR20] Grzanka PR, Brian JD, Shim JK (2016). My Bioethics Will Be Intersectional or It Will Be [Bleep]. The American Journal of Bioethics.

[CR21] Harford J (2018). The perspectives of women professors on the professoriate: A missing piece in the narrative on gender equality in the university. Education Sciences.

[CR22] Herrmann, V., E. Zafarana, M. Aksözen, and M. Bajic. 2019. Gender policy monitoring in Swiss higher education institutions. UNECE Conference of European Statisticians, Neuchatel. https://slidetodoc.com/gender-policy-monitoring-in-swiss-higher-education-institutions/. Accessed September 21, 2021.

[CR23] Holter, Ø.G., H. Svare, and C. Egeland. 2009. *Gender Equality and Quality of Life. A Norwegian Perspective.* Oslo: The Nordic Gender Institute.

[CR24] Klugman C. 2017. The aftermath of Charlottesville: What’s a bioethicist to do? *Bioethics Today*, August 18. https://bioethicstoday.org/blog/the-aftermath-of-charlottesville-whats-a-bioethicist-to-do/. Accessed September 21, 2021.

[CR25] Kreissl K, Streidinger A, Sauer B, Hofbauer J (2015). Will gender equality ever fit in? Contested discursive spaces of university reform. Gender and Education..

[CR26] Krzaklewska, E. 2014. Measurement of gender equality—Analysing dimensions, embracing areas, considering contexts. Working Paper No. 1.2 Gender Equality and Quality of Life—State of Art Report. Jagiellonian University of Krakow. 1–33.

[CR27] Leybold-Johnson, I. 2017. Why talented women are disappearing from Swiss universities. Swissinfo. https://www.swissinfo.ch/eng/young-female-academics_why-talented-women-are-disappearing-from-swiss-universities/43103010. Accessed September 21, 2021.

[CR28] Mayes C, Paradies Y, Elias A (2021). Lead essay—institutional racism, whiteness, and the role of critical bioethics. Journal of Bioethical Inquiry.

[CR29] Moffitt, U. 2020. Germany is not the anti-racist model we’re looking for. *Medium*, July 13.

[CR30] Myser C (2003). Differences from somewhere: The normativity of whiteness in bioethics in the United States. American Journal of Bioethics.

[CR31] Nentwich J. 2006. Equity and diversity at universities in Switzerland. https://www.alexandria.unisg.ch/34848/1/06_Nentwich_DiversityUniversity.pdf Accessed September 21, 2021.

[CR32] Neyer G, Lappegård T, Vignoli D (2013). Gender equality and fertility: Which equality matters?. European Journal of Population / Revue Européenne de Démographie.

[CR33] Oltermann, P., and J. Henley. 2020. France and Germany urged to rethink reluctance to gather ethnicity data. *The Guardian*, June 16.

[CR34] Pellert A, Gindl M, Sagaria M (2007). Gender equity and higher education reform in Austria. Women, universities and change: Gender equality in the European Union and the United States.

[CR35] Peterson H (2015). Exit the king, enter the maid: Changing discourses on gendered management ideals in Swedish higher education. Gender in Management.

[CR36] Peterson, H., and B. Jordansson. 2017. Gender equality as a core academic value: Undoing gender in a “non-traditional” Swedish university. In *Gendered Success in Higher Education*, edited by K. White and P. O’Conner.

[CR37] Powell S, Ah-King M, Hussénius A (2018). “Are we to become a gender university?” Facets of resistance to a gender equality project. Gender, Work and Organization..

[CR38] Ray, K. 2020. Black bioethics and how the failures of the profession paved the way for its existence. The Hastings Center. https://www.thehastingscenter.org/black-bioethics-and-how-the-failures-of-the-profession-paved-the-way-for-its-existence/. Accessed September 21, 2021.

[CR39] Rayes, D. 2021. Faith and mental health help shape the integration of Muslim refugees in Germany. *Migration Policy Institute*. https://www.migrationpolicy.org/article/faith-mental-health-integration-muslim-refugees-germany. Accessed September 21, 2021.

[CR40] Truong M, Sharif MZ (2021). We’re in this together: A reflection on how bioethics and public health can collectively advance scientific efforts towards addressing racism. Journal of Bioethical Inquiry.

[CR41] van den Brink M, Benschop Y (2012). Slaying the seven-headed dragon: The quest for gender change in academia. Gender, Work and Organization..

[CR42] Wilson YY (2021). Bioethics, race, and contempt. Journal of Bioethical Inquiry.

[CR43] World Health Organization. 2022. Health topics: Gender. https://www.euro.who.int/en/health-topics/health-determinants/gender/gender-definitions. Accessed on March 7, 2022.

[CR44] Wroblewski A, Leitner A (2011). Equal opportunities policies at Austrian universities and their evaluation: Development, results and limitations. Brussels Economic Review.

[CR45] Wroblewski A., and A. Striedlinger. 2018. Gender equality in science and research in Austria. *Austrian Federal Ministry of Education, Science and Research*.

