# Supplementary material for: Diversity in German-speaking medical ethics and humanities
Source: J Bioeth Inq. 2022 Nov 7;19(4):643–53. doi: 10.1007/s11673-022-10215-6 (PMC9908651; doi:10.1007/s11673-022-10215-6)
Supplement: Supplementary file 1 — Supplementary file1 (DOCX 39 KB) [file 11673_2022_10215_MOESM1_ESM.docx]

## **Supplemental Material**

## **Table 1** Gender diversity in German institutes

| **No.** | **Institution** | **Staff Position** | | | | | **Total** | **Staff gender** | | **Chi-square** |
| --- | --- | --- | --- | --- | --- | --- | --- | --- | --- | --- |
|  |  | **Director** | **Researchers and lecturers** | **Associated**  **researchers** | **Student assistants** | **Admin** |  |  |  |  |
|  |  |  |  |  |  |  |  | **Male**  **n (%)** | **Female**  **n (%)** |  |
| **Aachen** | | | | | | | | | |  |
| 1 | Institut für Geschichte, Theorie und Ethik der Medizin, Universitätsklinikum Aachen  Director: Dominik Groß | 1  (1.8) | 10  (17.5) | 37  (64.9) | 7  (12.3) | 2  (3.5) | **57**  **(100)** | 29  (50.9) | 28  (49.1) | χ^2^_(1)_=.02, *P=*.89 |
| **Berlin** | | | | | | | | | |  |
| 2 | Institut für Geschichte der Medizin und Ethik in der Medizin, Charité Berlin - Universitätsmedizin Berlin  Director: Volker Hess | 1  (3.3) | 19  (63.3) | 8  (26.7) | 0 | 2  (6.7) | **30**  **(100)** | 12  (40) | 18  (60) | χ^2^_(1)_=1.2, *P=*.27 |
| 3 | Research Group Strech, Berlin Institute of Health  QUEST Center  Director: Daniel Strech | 1  (5.3) | 15  (78.9) | 0 | 2  (10.5) | 1  (5.3) | **19**  **(100)** | 8  (42.1) | 11  (57.9) | χ^2^_(1)_=.47, *P=*.49 |
| **Bochum** | | | | | | | | | |  |
| 4 | Institut für Medizinische Ethik und Geschichte der Medizin, Ruhr-Universität Bochum  Director: Jochen Vollmann | 1  (5) | 11  (55) | 0 | 6  (30) | 2  (10) | **20**  **(100)** | 9  (45) | 11  (55) | χ^2^_(1)_=.20, *P=*.65 |
| **Bonn** | | | | | | | | | |  |
| 5 | Institute for Medical Humanities,  Universität Bonn  Director: Mariacarla Gadebusch Bondio | 1  (5.9) | 6  (35.3) | 3  (17.6) | 4  (23.5) | 3  (17.6) | **17**  **(100)** | 9  (52.9) | 8  (47.1) | χ^2^_(1)_=.06, *P=*.81 |
| 6 | Deutsches Referenzzentrum für Ethik in den Biowissenschaften  Director: Dieter Sturma | 1  (5.9) | 5  (29.4) | 0 | 4  (23.5) | 7  (41.2) | **17**  **(100)** | 7  (41.2) | 10  (58.8) | χ^2^_(1)_=.53, *P=*.47 |
| **Dresden** | | | | | | | | | |  |
| 7 | Institut für Geschichte der Medizin, Technische Universität Dresden  Director: Caris-Petra Heidel | 1  (11.1) | 2  (22.2) | 3  (33.3) | 0 | 3  (33.3) | **9**  **(100)** | 2  (22.2) | 7  (77.8) | χ^2^_(1)_=2.8, *P=*.10 |
| **Düsseldorf** | | | | | | | | | |  |
| 8 | Institut für Geschichte, Theorie und Ethik der Medizin, Universitätsklinikum Düsseldorf  Director: Heiner Fangerau | 1  (2.4) | 25  (61) | 4  (9.8) | 8  (19.5) | 3  (7.3) | **41**  **(100)** | 20  (48.8) | 21  (51.2) | χ^2^_(1)_=.02, *P=*.88 |
| **Erlangen - Nürnberg** | | | | | | | | | |  |
| 9 | Institut für Geschichte und Ethik der Medizin, Friedrich-Alexander-Universität Erlangen-Nürnberg  Director: Karl-Heinz Leven | 1  (1.8) | 17  (30.4) | 30  (53.6) | 6  (10.7) | 2  (3.6) | **56**  **(100)** | 20  (35.7) | 36  (64.3) | **χ^2^_(1)_=4.6, *P=*.03** |
| 10 | Lehrstuhl für Systematische Theologie II (Ethik), Friedrich-Alexander-Universität Erlangen-Nürnberg  Director: Peter Dabrock | 1  (6.7) | 4  (26.7) | 0 | 9  (60) | 1  (6.7) | **15**  **(100)** | 5  (33.3) | 10  (66.7) | χ^2^_(1)_=1.7, *P=*.20 |
| **Frankfurt/Main^1^** | | | | | | | | | |  |
| 11 | Institut für Geschichte und Ethik der Medizin, Johann Wolfgang Goethe Universität Frankfurt am Main  Director: Udo Benzenhöfer | 1  (11.1) | 6  (66.7) | 0 | 0 | 2  (22.2) | **9**  **(100)** | 3  (33.3) | 6  (66.7) | χ^2^_(1)_=1.0, *P=*.32 |
| **Freiburg** | | | | | | | | | |  |
| 12 | Institut für Ethik und Geschichte der Medizin, Albert-Ludwigs-Universtität Freiburg  Director: Giovanni Maio | 1  (16.7) | 1  (16.7) | 4  (66.7) | 0 | 0 | **6**  **(100)** | 5  (83.3) | 1  (16.7) | χ^2^_(1)_=2.7, *P=*.10 |
| **Gießen** | | | | | | | | | |  |
| 13 | Institut für Geschichte der Medizin, Justus-Liebig-Universität Gießen  Director: Volker Roelcke | 1  (7.7) | 4  (30.8) | 4  (30.8) | 0 | 4  (30.8) | **13**  **(100)** | 7  (53.8) | 6  (46.2) | χ^2^_(1)_=.10, *P=*.78 |
| **Göttingen** | | | | | | | | | |  |
| 14 | Instituts für Ethik und Geschichte der Medizin, Universitätsmedizin Göttingen  Director: Claudia Wiesemann | 1  (3.1) | 14  (43.8) | 17  (53.1) | 0 | 0 | **32**  **(100)** | 11  (34.4) | 21  (65.6) | χ^2^_(1)_=3.1, *P=*.08 |
| **Greifswald** | | | | | | | | | |  |
| 15 | Institut für Ethik und Geschichte der Medizin, Universitätsmedizin Greifswald  Director: Hartmut Bettin | 1  (16.7) | 3  (50) | 0 | 0 | 2  (33.3) | **6**  **(100)** | 2  (33.3) | 4  (66.7) | χ^2^_(1)_=.67, *P=*.41 |
| **Halle/Saale** | | | | | | | | | |  |
| 16 | Institut für Geschichte und Ethik der Medizin, Martin-Luther-Universität Halle-Wittenberg  Director: Jan Schildmann | 1  (4.8) | 10  (47.6) | 0 | 8  (38.1) | 2  (9.5) | **21**  **(100)** | 10  (47.6) | 11  (52.4) | χ^2^_(1)_=.05, *P=*.83 |
| **Hamburg** | | | | | | | | | |  |
| 17 | Institut für Geschichte und Ethik der Medizin Universitätsklinikum Hamburg  Director: Philipp Osten | 1  (7.1) | 9  (64.3) | 2  (14.3) | 0 | 2  (14.3) | **14**  **(100)** | 6  (42.9) | 8  (57.1) | χ^2^_(1)_=.30, *P=*.59 |
| **Hagen** | | | | | | | | | |  |
| 18 | Juniorprofessur für Medizinethik, Institut für Philosophie,  FernUniversität in Hagen  Director: Orsolya Friedrich | 1  (12.5) | 4  (50) | 0 | 2  (25) | 1  (12.5) | **8**  **(100)** | 2  (25) | 6  (75) | χ^2^_(1)_=2.0, *P=*.16 |
| **Hannover** | | | | | | | | | |  |
| 19 | Institut für Geschichte, Ethik und Philosophie der Medizin, Medizinische Hochschule Hannover  Director: Sabine Salloch | 1  (3.3) | 17  (56.7) | 3  (10) | 7  (23.3) | 2  (6.7) | **30**  **(100)** | 14  (46.7) | 16  (53.3) | χ^2^_(1)_=.13, *P=*.72 |
| 20 | Zentrum für Gesundheitsethik, Evangelisch-lutherischen Landeskirche Hannovers  Director: Julia Inthorn | 1  (14.3) | 3  (42.9) | 0 | 1  (14.3) | 2  (28.6) | **7**  **(100)** | 2  (28.6) | 5  (71.4) | χ^2^_(1)_=1.3, *P=*.26 |
| **Heidelberg** | | | | | | | | | |  |
| 21 | Institut für Geschichte und Ethik der Medizin, Ruprecht-Karls-Universität Heidelberg  Director: Karen  Nolte | 1  (3.8) | 12  (46.2) | 1  (3.8) | 10  (38.5) | 2  (7.7) | **26**  **(100)** | 10  (38.5) | 16  (61.5) | χ^2^_(1)_=1.4, *P=*.24 |
| 22 | Ethics and Patient oriented Care in Oncology (NCT-EPOC), Nationalen Centrums für Tumorerkrankungen (NCT) Heidelberg  Director: Eva Winkler | 1  (5.3) | 13  (68.4) | 1  (5.3) | 4  (21.1) | 0 | **19**  **(100)** | 8  (42.1) | 11  (57.9) | χ^2^_(1)_=.47, *P=*.49 |
| **Jena** | | | | | | | | | |  |
| 23 | Institut für Geschichte, Theorie und Ethik der Medizin, Friedrich-Schiller-Universität Jena  Director: Nikolaus Knoepffler | 1  (10) | 4  (40) | 2  (20) | 0 | 3  (30) | **10**  **(100)** | 5  (50) | 5  (50) | χ^2^_(1)_=.000, *P=*1.0 |
| **Kiel** | | | | | | | | | |  |
| 24 | Institut für Experimentelle Medizin - Geschäftsbereichs der Medizinethik, Christian-Albrechts-Universität zu Kiel  Director: Claudia Bozzaro | 1  (16.7) | 4  (66.7) | 0 | 0 | 1  (16.7) | **6**  **(100)** | 1  (16.7) | 5  (83.3) | χ^2^_(1)_=2.7, *P=*.10 |
| **Köln** | | | | | | | | | |  |
| 25 | Institut für Geschichte und Ethik der Medizin (History), Universität Köln  Director: Axel Karenberg | 1  (6.3) | 9  (56.3) | 1  (6.3) | 3  (18.8) | 2  (12.5) | **16**  **(100)** | 12  (75) | 4  (25) | **χ^2^_(1)_=4.0, *P=*.05** |
| 26 | Institut für Geschichte und Ethik der Medizin (Ethics) / CERES – Cologne Center for ethics, rights, economics, and social sciences of health, Universität Köln  Director: Christiane Woopen | 1  (2) | 30  (58.8) | 5  (9.8) | 12  (23.5) | 3  (5.9) | **51**  **(100)** | 12  (23.5) | 39  (76.5) | **χ^2^_(1)_=14.3, *P*<.001** |
| **Leipzig** | | | | | | | | | |  |
| 27 | Institut für Geschichte der Medizin und Naturwissenschaften, Universität Leipzig  Director: Ortrun Riha | 1  (20) | 2  (40) | 1  (20) | 0 | 1  (20) | **5**  **(100)** | 1  (20) | 4  (80) | χ^2^_(1)_=1.8, *P=*.18 |
| **Lübeck** | | | | | | | | | |  |
| 28 | Institut für Medizingeschichte und Wissenschaftsforschung, Universität zu Lübeck  Director: Cornelius Borck | 1  (5.3) | 14  (73.7) | 0 | 0 | 4  (21.1) | **19**  **(100)** | 10  (52.6) | 9  (47.4) | χ^2^_(1)_=.05, *P=*.82 |
| **Magdeburg** | | | | | | | | | |  |
| 29 | Institut für Geschichte, Ethik und Theorie der Medizin, Otto-von-Guericke-Universität Magdeburg  Director: Eva Brinkschulte | 1  (16.7) | 3  (50) | 1  (16.7) | 0 | 1  (16.7) | **6**  **(100)** | 2  (33.3) | 4  (66.7) | χ^2^_(1)_=.67, *P=*.41 |
| **Mainz** | | | | | | | | | |  |
| 30 | Institut für Geschichte, Theorie und Ethik der Medizin, Universitätsmedizin der Johannes Gutenberg Universität Mainz  Director: Norbert W. Paul | 1  (5.3) | 4  (21.1) | 3  (15.8) | 7  (36.8) | 4  (21.1) | **19**  **(100)** | 9  (47.4) | 10  (52.6) | χ^2^_(1)_=.05, *P=*.82 |
| **Mannheim** | | | | | | | | | |  |
| 31 | Institut für Geschichte, Theorie und Ethik der Medizin, Universitätsmedizin Mannheim  Director: Axel W. Bauer | 1  (20) | 2  (40) | 0 | 2  (40) | 0 | **5**  **(100)** | 3  (60) | 2  (40) | χ^2^_(1)_=.20, *P=*.66 |
| **Marburg** | | | | | | | | | |  |
| 32 | Arbeitsstelle für Geschichte der Medizin, Philipps-Universität Marburg  Director: Tanja Pommerening | 1  (16.7) | 2  (33.3) | 2  (33.3) | 0 | 1  (16.7) | **6**  **(100)** | 2  (33.3) | 4  (66.7) | χ^2^_(1)_=.67, *P=*.41 |
| **München** | | | | | | | | | |  |
| 33 | Institut für Geschichte und Ethik der Medizin, Technische Universität München  Director: Alena M. Buyx | 1  (3.4) | 16  (55.2) | 2  (6.9) | 5  (17.2) | 5  (17.2) | **29**  **(100)** | 12  (41.4) | 17  (58.6) | χ^2^_(1)_=.86, *P=*.35 |
| 34 | Institut für Ethik, Geschichte und Theorie der Medizin, Ludwig-Maximilians-Universität München  Director: Georg Marckmann | 1  (2.9) | 15  (44.1) | 11  (32.4) | 5  (14.7) | 2  (5.9) | **34**  **(100)** | 17  (50) | 17  (50) | χ^2^_(1)_=.000, *P=*1.0 |
| **Münster** | | | | | | | | | |  |
| 35 | Institut für Ethik, Geschichte und Theorie der Medizin, Westfälischen Wilhelms-Universität Münster  Director: Bettina Schöne-Seifert | 1  (5) | 9  (45) | 8  (40) | 0 | 2  (10) | **20**  **(100)** | 14  (70) | 6  (30) | χ^2^_(1)_=3.2, *P=*.07 |
| **Oldenburg** | | | | | | | | | |  |
| 36 | Carl von Ossietzky Universität Oldenburg  Department für Versorgungsforschung -Abteilung Medizinische Ethik  Director: Mark Schweda | 1  (14.3) | 5  (71.4) | 0 | 1  (14.3) | 0 | **7**  **(100)** | 4  (57.1) | 3  (42.9) | χ^2^_(1)_=.14, *P=*.71 |
| **Rostock** | | | | | | | | | |  |
| 37 | Universitätsmedizin Rostock  Arbeitsbereich Geschichte der Medizin  Director: Ekkehardt Kumbier | 1  (12.5) | 6  (75) | 0 | 1  (12.5) | 0 | **8**  **(100)** | 2  (25) | 6  (75) | χ^2^_(1)_=2.0, *P=*.16 |
| **Tübingen** | | | | | | | | | |  |
| 38 | Universität Tübingen  Institut für Ethik und Geschichte der Medizin  Director: Urban Wiesing | 1  (5.3) | 10  (52.6) | 0 | 4  (21.1) | 4  (21.1) | **19**  **(100)** | 10  (52.6) | 9  (47.4) | χ^2^_(1)_=.05, *P=*.82 |
| **Ulm** | | | | | | | | | |  |
| 39 | Universität Ulm  Institut für Geschichte, Theorie und Ethik der Medizin  Director: Florian Steger | 1  (2.4) | 27  (65.9) | 0 | 10  (24.4) | 3  (7.3) | **41**  **(100)** | 24  (58.5) | 17  (41.5) | χ^2^_(1)_=1.2, *P=*.27 |
| **Vallendar** | | | | | | | | | |  |
| 40 | Lehrstuhl für Ethik, Theorie und Geschichte der Medizin, Philosophisch-Theologische Hochschule Vallendar (PTHV)  Director: Thomas Heinemann | 1  (100) | 0 | 0 | 0 | 0 | **1**  **(100)** | 1  (100) |  | N/A |
| 41 | Ethik-Institut, Philosophisch-Theologische Hochschule Vallendar (PTHV)  Director: Ingo Proft | 1  (33.3) | 1  (33.3) | 0 | 1  (33.3) | 0 | **3**  **(100)** | 1  (33.3) | 2  (66.7) | χ^2^_(1)_=.33, *P=*.56 |
| **Würzburg** | | | | | | | | | |  |
| 42 | Universität Würzburg  Institut für Geschichte der Medizin  Director: Michael Stolberg | 1  (4.2) | 7  (29.2) | 10  (41.7) | 4  (16.7) | 2  (8.3) | **24**  **(100)** | 18  (75) | 6  (25) | **χ^2^_(1)_=6.0, *P=*.01** |
|  | | | | | | | | | | |
| **Total**  **N (%)** |  | 42  (5.2) | 380  (47.4) | 163  (20.3) | 133  (16.6) | 83  (10.4) | **801**  **(100)** | 361  (45.1) | 440  (54.9) | **χ^2^_(1)_=7.8, *P=*.005** |

^1^ On March 26, 2021, it was announced by the German Academy of Ethics in Medicine that Prof. Udo Benzenhöfer, Director of the Goethe-Universität Frankfurt am Main´s Instituts für Geschichte und Ethik der Medizin, had passed away.
