# Supplementary material for: Diversity in German-speaking medical ethics and humanities
Source: J Bioeth Inq. 2022 Nov 7;19(4):643–53. doi: 10.1007/s11673-022-10215-6 (PMC9908651; doi:10.1007/s11673-022-10215-6)
Supplement: Supplementary file 2 — Supplementary file2 (DOCX 17 KB) [file 11673_2022_10215_MOESM2_ESM.docx]

## **Supplemental Material**

## **Table 2** Gender diversity in Swiss German institutes

| **Nr.** | **Institution** | **Staff Position** | | | | | **Total** | **Staff gender** | | **Chi-square** |
| --- | --- | --- | --- | --- | --- | --- | --- | --- | --- | --- |
|  |  | **Director** | **Researchers and lecturers** | **Associated**  **researchers** | **Student assistants** | **Admin** |  |  |  |  |
|  |  |  |  |  |  |  |  | **Male**  **n (%)** | **Female**  **n (%)** |  |
| **Basel** | | | | | | | | | |  |
| 1 | Institute of Biomedical Ethics, University of Basel  Director: Bernice Elger | 1  (2.3) | 23  (53.5) | 18  (41.9) | 0 | 1  (2.3) | **43**  **(100)** | 20  (46.5) | 23  (53.5) | χ^2^_(1)_=.21, *P=*.65 |
| **Bern** | | | | | | | | | |  |
| 2 | Institut für Medizingeschichte, Universität Bern  Director: Hubert Steinke | 1  (4.3) | 9  (39.1) | 10  (43.5) | 1  (4.3) | 2  (8.7) | **23**  **(100)** | 16  (69.6) | 7  (30.4) | χ^2^_(1)_=3.5, *P=*.06 |
| 3 | Leitung Medizinethik und ärztliche Weiterbildung, Universitätsspital Bern  Director: Rouven Porz | 1  (50) | 1  (50) | 0 | 0 | 0 | **1**  **(100)** | 1  (100) | 0 | N/A |
| **Zürich** | | | | | | | | | |  |
| 4 | Institut für Biomedizinische Ethik und Medizingeschichte (IBME), Universität Zürich  Director: Nikola Biller-Andorno | 1  (1.4) | 40  (55.6) | 26  (36.1) | 0 | 5  (6.9) | **72**  **(100)** | 25  (34.7) | 47  (65.3) | **χ^2^_(1)_=6.7, *P=*.01** |
| 5 | Chair of Bioethics, ETH Zürich  Director: Effy Vayena | 1  (7.1) | 7  (50) | 5  (35.7) | 0 | 1  (7.1) | **14**  **(100)** | 6  (42.9) | 8  (57.1) | χ^2^_(1)_=.29, *P=*.59 |
|  | | | | | | | | | | |
| **Total**  **N (%)** |  | 5  (3.3) | 80  (51.9) | 59  (38.3) | 1  (0.6) | 9  (5.8) | **154**  **(100)** | 69  (44.8) | 85  (55.2) | χ^2^_(1)_=1.6, *P=*.20 |
