# Supplementary material for: Diversity in German-speaking medical ethics and humanities
Source: J Bioeth Inq. 2022 Nov 7;19(4):643–53. doi: 10.1007/s11673-022-10215-6 (PMC9908651; doi:10.1007/s11673-022-10215-6)
Supplement: Supplementary file 3 — Supplementary file3 (DOCX 15 KB) [file 11673_2022_10215_MOESM3_ESM.docx]

## **Supplemental Material**

## **Table 3** Gender diversity in Austrian institutes

| **Nr.** | **Institution** | **Staff position** | | | | | **Total** | **Staff gender** | | **Chi-square** |
| --- | --- | --- | --- | --- | --- | --- | --- | --- | --- | --- |
|  |  | **Director** | **Researchers and lecturers** | **Associated**  **researchers** | **Student assistants** | **Admin** |  |  |  |  |
|  |  |  |  |  |  |  |  | **Male**  **n (%)** | **Female**  **n (%)** |  |
| **Vienna** | | | | | | | | | |  |
| 1 | Ethik, Sammlungen und Geschichte der Medizin, Medizinische Universität Wien  Director: Christiane Druml | 1  (100) | 0 | 0 | 0 | 0 | **1**  **(100)** | 0 | 1  (100) | N/A |
| **Innsbruck** | | | | | | | | | |  |
| 2 | Austrian Unit of the Network of Institutions for Medical Ethics Education, Medizinische Universität Innsbruck |  |  | 8  (100) |  |  | **8**  **(100)** | 4  (50) | 4  (50) | χ^2^_(1)_=.000, *P=*1.0 |
|  | | | | | | | | | | |
| **Total**  **N(%)** |  | 1  (11.1) |  | 8  (88.9) |  |  | **9**  **(100)** | 4  (44.4) | 5  (55.6) | χ^2^_(1)_=.11, *P=*.74 |
